# Dignity of older home-dwelling women nearing end-of-life: Informal caregivers’ perception

**DOI:** 10.1177/0969733020956372

**Published:** 2020-10-28

**Authors:** Katrine Staats, Ellen Karine Grov, Bettina S. Husebø, Oscar Tranvåg

**Affiliations:** 1658 University of Bergen, Norway; 60499 Oslo Metropolitan University, Norway; 1658 University of Bergen, Norway; 1657 Western Norway University of Applied Sciences, Norway; Oslo University Hospital, Norway

**Keywords:** Areas of practice, care of the older person, dignity in care, empirical approaches, end of life issues, home care, palliative care, qualitative research, topic areas

## Abstract

**Background:**

Most older people wish to live in the familiar surroundings of their own home until they die. Knowledge concerning dignity and dignity loss of home-dwelling older women living with incurable cancer should be a foundation for quality of care within municipal healthcare services. The informal caregivers of these women can help increase the understanding of sources related to dignity and dignity loss

**Aim:**

The aim of this study was to explore informal caregivers’ perceptions of sources related to dignity and dignity loss in end-of-life of older home-dwelling women with incurable cancer.

**Research design and method:**

The study was founded upon Gadamer’s philosophical hermeneutics. In-depth interviews with 13 informal caregivers were carried out, and four participant observations were performed during home meetings.

**Ethical consideration:**

The study was based on voluntary participation, informed consent, confidentiality and the opportunity to withdraw at any time. The Norwegian Social Science Data Services approved the study.

**Results:**

Three main sources important in preserving the older women’s dignity were identified: maintaining one’s self-concept, remaining hopeful and sustaining freedom of choice. We also identified three main sources that lead to dignity loss: Sensing loss of human value, experiencing absence of gentleness and feelings of being treated as an object.

**Discussion and final considerations:**

On the individual level, the opportunity to maintain one’s self-concept and control in life, preserved dignity, while feelings of existential loneliness led to dignity loss. On the relational level, being confirmed as worthy human beings promoted the women’s dignity, whereas dignity loss was related to uncaring behaviours from healthcare professionals. On the societal level, individual decisions concerning travel situations and the place to stay when nearing end-of-life were of crucial importance. Constituting these women’s living space, these perspectives should be emphasized in healthcare professionals’ educational training and in the municipal end-of-life care of these patients.

## Introduction

The concept of dignity is important in the context of end-of-life care and has a major influence on the quality of care given by informal caregivers (ICs) and healthcare professionals (HCPs).^1^ The United Nations has focused attention on human dignity and dignity-preservation stating that all human beings have an inherent dignity – thus having one’s dignity preserved is a fundamental human right.^2^ Furthermore, the World Health Organization claims that all people have the right to be treated with dignity and stresses that women are more likely to experience dignity-degrading treatments and practices due to the heritage of traditional and disadvantageous gender roles.^3^ In Norway, regulations concerning dignified care for older people aims to guarantee older people a dignified, safe and meaningful life.^4^ Internationally there seems to be an agreement that dignity and dignifying care are important within healthcare services.^5^

Being diagnosed with cancer is strongly related to age and a major cause of mortality among individuals 65 years and older.^6^ Worldwide, the cancer incidence for women was estimated to be 8.5 million cases in 2018.^7^ Women live longer than men and generally experience poorer health.^8^ In addition, many older women outlive their spouses and live alone with an increased risk of social isolation.^9^

Informal caregivers provide most of the care and practical support for older people.^10^ However, older women living with incurable cancer seem to have less support from family members compared to men^11^ and make less use of municipal services.^12^ Informal caregiving can be perceived as a meaningful and appreciated task, but can also be a highly stressful experience accompanied by a strong ethical commitment to not let their loved ones down.^13^

The opportunities to receive professional end-of-life care at home vary.^14^ Most people wish to spend their last days of life in familiar surroundings – and to die there.^15^ In Norway, the majority of these patients become nursing home residents in the last phase of life.^16^ Only 15% of people who died in Norway in 2013 died at home, and of these only 6.3% had planned a home-death.^17^ However, end-of-life care seemed to be of a higher quality and more dignifying when given at home compared to institutional care.^1,18^

In a previous study^19^, we documented how older women with incurable cancer perceived having control, experiencing hope and meaningfulness and feeling valued as human beings, to be crucial sources for preserving dignity. We also described how losing self-determination, experiencing violation of their personal lives and feeling worthless, all led to dignity loss. However, we know little about what ICs close to these female patients perceive as crucial sources in preserving dignity, and which sources promote dignity loss. This proxy perspective would be a valuable contribution to the existing knowledge and increases the foundation for developing a dignity-preserving end-of-life care practice within the municipal healthcare services.

## Aim

The aim of this study was to explore ICs perceptions of sources related to dignity and dignity loss of home-dwelling older women with incurable cancer nearing end-of-life.

## Methodology

Gadamer’s hermeneutical methodology was used in which interpretation of texts is fundamental in developing an understanding of their meanings.^20^ We emphasized the process of hermeneutic circle movements to move beyond our pre-understanding, towards developing *new* understanding, while being oriented towards the project’s aim.^20^

### Participants and setting

Oncology coordinators in 11 Norwegian municipalities assisted with participant recruitment. The following inclusion criteria were used: ICs having at least weekly contact and responsibility for informal end-of-life care for a woman aged 65 years or older, who lives with incurable cancer at home, and is currently receiving municipal healthcare services. During the recruitment period, from November 2018 to December 2019, 23 ICs were asked to participate in the study. Of these, 10 participants declined due to tiredness, less involvement in daily care, or suddenly becoming bereaved. In all, 13 ICs, 7 men and 6 women aged 40–77, gave their consent to participate (Table 1). In addition, 4 of the 13 interviewed IC’s gave their consent to take part in participant observations. The women with cancer and their HCP also consented to participate. The first author (K.S.) conducted participant observation of three home-meetings and one meeting at the hospital.

**Table 1.** Participants’ age and relation to the older women with cancer.

Participant	Age	Relation
1	67	Husband
2	70	Husband
3	77	Husband
4	74	Husband
5	42	Daughter
6	45	Son
7	65	Sister
8	40	Daughter
9	74	Husband
10	45	Daughter
11	59	Daughter
12	73	Husband
13	66	Sister

### Informal caregiver representatives as co-researchers

We acknowledge both patients and IC representatives as experts with unique competence, valuable in all phases of the research process. Inspired by the Framework of patient and IC participation in research (PAICPAIR part 1)^21^ we established contact with patient organizations in the planning phase of this research process to create a sound collaboration with ICs as co-researchers. Two ICs were recruited as co-researchers. They had previously been ICs in the end-of-life of a home-dwelling woman, aged 65 or older, with incurable cancer. They became IC representatives in the project steering group and advisory board, respectively. Other co-researchers included a home-dwelling woman (over 65 years old) with incurable cancer, a medical doctor, two oncology nurses and two research advisors – all with end-of-life care experiences. They provided us with constructive feedback on our initial project ideas, the research questions and interview guide. They brought nuances from their experiences into the study, which increased the quality of our data collection.^21^ We maintained contact with the ICs by offering home visits and phone calls instead of expecting them to read several documents via email. We also kept in contact by digital newsletters describing the project’s development. In addition, both ICs participated in discussions of preliminary findings, were engaged in the ongoing evaluation of the overall project process, and will be involved in disseminating the study results.^21^

### Data collection

We used individual in-depth interviews^22^ and participant observations^23^ as data collection tools. A semi-structured, modifiable interview guide was developed to structure the data collection conversation. In line with the hermeneutical methodology,^20^ exploration of new themes and reconstructions of the interview guide appeared throughout the data collection process. Examples of questions guiding the interviews were: *Can you describe a situation, after your wife/mother/sister got sick, as an example of dignity preserving care? Alternatively, a situation leading to dignity loss?* The first author (K.S.) conducted the interviews, all of them were recorded and transcribed verbatim. One interview lasting between 55 and 81 min (mean = 68.6 min) was performed with each participant, producing 220 transcribed A4 pages for data interpretation. Data collection also consisted of 16 pages of field notes from four participant observations (Table 2). The first author observed the interaction between ICs, the older women with cancer and HCP responsible for municipal end-of-life care at home. The participant observations lasted from 50 min to 2 h (mean = 82.5 min). Utilizing an observation guide, crucial care-related aspects that preserved dignity, and those leading to dignity loss, were observed and noted. Examples of themes guiding the observations were: *Which sources seem to contribute to the ill women’s experiences of dignity, when observing the interaction between the women, IC’s and HCPs? How do HCP communicate and interact with the ill women when she is sharing degrading experiences?* In this context, informal conversations were also carried out to get a better understanding of the observations. In addition, language, voice sound, important comments, key-phrases and passages were noted and guided the focus of the participant observations.

**Table 2.** Participant observation − study participants, HCPs and setting.

Observation	Participants observed	Setting
1	Patient, husband, cancer coordinator, palliative-care doctor, observer	Patient’s home
2	Patient, husband, cancer coordinator, observer	Patient’s home
3	Patient, husband, palliative-care doctor and oncology nurse, observer	Hospital
4	Patient, husband, oncology nurse, observer	Patient’s home

### Interpretation

The empirical data were interpreted using Gadamer’s hermeneutical methodology.^20^ During the interpretation process, the authors met regularly to discuss preliminary emerging patterns of meaning. Understanding was achieved through a circular interpretive process that Gadamer called the hermeneutical circle, in which we moved from a preliminary interpretive understanding of each individual interview text, to an initial interpretation of the text as a whole. At this point, our preliminary new understanding helped us interpret and understand the various parts of the text as an interrelated, integrated whole, making it possible for us to formulate our final interpretive understanding. The intention of this hermeneutical process was to seek the truth according to Gadamer’s outline, and see beyond what was close at hand.^20^ Katie Eriksson’s *Theory of Caritative Caring*,^24,25^ in which safeguarding the dignity of the suffering human being is the ultimate goal, was chosen as the framework for the empirical-theoretical reflections performed in the discussion section, below.

### Pre-understanding

As an oncology nurse and researcher, first author K.S. was experienced in communicating with ICs concerning their present life situations. O.T. is a mental health nurse, E.K.G. an oncology nurse and B.S.H. a medical doctor, all three experienced researchers. Our pre-understanding is not neutral and distanced, but influenced by a committed relationship to the subject under investigation. As a research team we believed that ICs would perceive staying in familiar surroundings as a crucial dignity-preserving source, as well as a way to protecting the older women from their most illness-related burdens. Simultaneously, we assumed that ICs would describe that dignity of these older women may be affected by formal healthcare structures leading to situations in which the women received care from a large number of HCPs, as well as being exposed to unstructured planning in transitions.

### Ethical consideration

When recruiting IC’s of older women living with incurable cancer at home, ethical demands for researchers sensitivity were raised to protect the IC’s dignity throughout the research process. They were given the time they needed to determine whether to participate or not, and time and place for the interviews and observations were determined based on the participants’ wishes. Most of the participants were familiar with the research project since their wife/mother/sister had participated in the first part of this project.^19^ When carrying out participant observations in the home of the women with cancer, first author K.S. strived to be a natural part of the setting, safeguarded the need for privacy and made use of previous experiences as an oncology nurse. The ICs were assured anonymity and confidentiality regarding adaption and presentation and publication of the data. They were also informed that they could withdraw from the study without giving any reasons. All participants signed informed consent forms. They were also informed verbally by the oncology coordinator, as well as by the researcher before the interviews and the participant observations were performed. The study was conducted in accordance with the Declaration of Helsinki^26^ and was approved by the Norwegian Centre for Research Data (ref. no. 138698).

## Results

We identified three main sources preserving the older women’s dignity: *maintaining one’s self-concept, remaining hopeful* and *sustaining freedom of choice*. We also identified three main sources leading to dignity loss: *sensing loss of human value, experiencing absence of gentleness* and *feelings of being treated as an object*.

### Sources related to dignity-preservation

#### Maintaining one’s self-concept

The informal caregivers highlighted the importance for the woman to maintain a sense of self-worth as a human being when nearing end-of-life. They expressed the importance of letting the woman be the main person in her own life, and to follow her lead. It was of crucial importance for the woman to be recognized as an independent individual still carrying her name and not be reduced to a random carrier of a diagnosis:

Don’t look at her like someone who is reduced to ‘an illness’. It is not only the disease defining her as the person she is. It’s that…you exist while having an illness.(1)

When living close to normality and searching for the bright spots in life, they were more able to uphold their senses of self-worth and personal integrity. Several IC’s expressed the importance of being positive and grabbing hold of everyday occurrences, such as making a meal, reading a book and meeting up with friends and family, without discussing illness-related matters.

#### Remaining hopeful

The informal caregivers found that remaining hope was essential to these women in their challenging and uncertain life situations. They described how the women found new meaning that helped relieve their suffering and strengthen their hope – for example, hope for new medications and treatments, even though the disease had reached an advanced stage:

She has already accepted that her life is going to an end. However, at the same time, she has not. She has a constantly need of being oriented concerning new medications. Therefore, she has a little hope for a dramatic turn. Not yet. Maybe later…because there is hope on…keep going on for several years to come…(12)

Other people’s presence and care were crucial sources to enhance the women’s senses of being secure and safeguarded. Being seen as the women they are, and *experiencing other people’s interest* in their life stories, were valuable dignity-preserving sources. Data from the participant observations added nuances to the interview data; when a HCPs took the time to become acquainted with the woman, they could show compassion and gentleness based on their knowledge of her preferences and wishes:

There was this nurse working the nights…she was visiting my mother every early morning. She wrote some words on a napkin: «Have a great day». Every morning she did that. I remember she did that the first morning, and my mother woke up and expressed «How nice!» Little things like that are so valuable: «They see me», she said. (8)

#### Sustaining freedom of choice

Several ICs pointed out the women’s need and willingness to tailor their own end-of -life care, despite having limited strength due to their illness. It was therefore crucial for them to know that other people respected them and their wishes. They were enjoying and cherishing the quality of life when staying at home, deciding how to arrange their days and maintaining social contacts. At the same time, many of the women had experienced admissions to institutions and found it peaceful. The IC expressed the importance of respecting the women's wishes concerning preferred place to stay nearing end-of-life:

I know she wants to stay at home, and she feels much better there. I think that means a lot to her, deciding where to be. Even though it feels unsafe for me…who am I to tell her not to be at home? (5)

### Sources related to dignity loss

#### Sensing loss of human value

The informal caregivers described situations where the women’s decline in health affected their self-perception, making them feel of less value. Living with a deteriorating body evoked the women’s feelings of meaninglessness and existential loneliness. A sister of an old woman nearing end-of-life expressed it like this:

I can see her body weakening. She wants to have control in her life and be part of everything happening around her. It’s her sense of dignity in life. However, I sense her vulnerability when she cries between the sheets during nighttime. Then she tells me she has these dark and painful thoughts…difficult taking about…(13)

The informal caregivers also described situations where the women felt insignificant, frustrated, often related to situations in which they felt unable to participate in daily chores, for example, when finding that self-care had become too exhausting, and they were dependent on others:

It is about her dignity. Not being able to take a shower herself anymore. That she must be hosed down with water in another room…I think she is very sorry, losing her sense of self-efficacy. I believe this is affecting her experience of dignity. She is after all an independent type of woman…(9)

#### Experiencing absence of gentleness

In certain situations, the women felt degraded rather than being met with consideration by HCPs. As perceived by ICs, the women had a need to manage ‘little things’ in their everyday lives, despite their reduced physical capacities. This need was strongly related to their requirement to be involved in decision-making processes, where at times their voices seemed to be unheard. ICs described how the women sometimes felt humiliated and invaded by HCPs. In fact, sometimes they felt their legal rights had been disregarded:

This nurse went into my mother’s room with the intention to give her the evening care, but my mother said: «No, I can do it myself». Then the nurse answered: «No, come over here, I am going to do it for you…It says here that I am going to give you the evening care». She was already on her way to drag my mum into the bathroom…My mother got furious and sad, and eventually she asked the nurse to leave. She felt violated and unsafe. (8)

In such circumstances, absence of HCPs gentleness prevailed in physical care settings. Feelings of humiliation and uncertainty emerged due to the women’s senses of being a burden to the HCPs, in addition to carrying many other unspoken worries. In these situations, the women had to mobilize all their strength to be seen, listened to and be taken seriously. The ICs felt that the life of the ill woman laid in the hands of others; being cases of little value, made her feel insignificant and subsidiary to others:

There was this young nurse, even so with strong opinions…obviously not very experienced. She meant that she had to use a certain big size of a needle when she inserted the cannula in my wife’s hand. The nurse wouldn’t listen to my wife when she argued for using a smaller one. She said it was not our business; needles were expensive and so on…She was pretty revolting and brutal: «You just have to bear with this» – so that was quite…shocking for her. (1)

#### Feelings of being treated as an object

HCPs willingness and effort to devote sufficient resources and spend sufficient time with the women were perceived as vital. The ICs described situations where the women felt unsafe and that they were regarded as objects more than people, for example, when changes and efficiency strategies within the healthcare system made them feel overlooked as individuals. This was also identified in our participant observations, where some of the women felt insignificant as the doctor in charge of their treatment seemed poorly prepared to follow up her specific treatment plan:

The doctor hadn’t even looked at the X-rays, he didn’t have the time…we did not get any information at all. Why should we come there at all then? It was quite a strain for my mother to get there. A lot of suffering when walking all the corridors as well as hours of nodding off in the waiting room because there is a lot of waiting…(5)

Different events seemed to have a negative impact on the lives of the women. This included, for example, unworthy and burdensome transportations between hospitals and their homes, where the women sometimes felt they were treated like objects while being in states of dependency. Several ICs referred to such negative experiences, where the women felt that they were not being acknowledged as individuals:

Most of the transportations are ‘collective journeys’. That is not very satisfying or dignifying when you are feeling ill. My wife had to sit four hours waiting for a taxi to be full enough before leaving…although 30 taxis were standing ready. It is not well paid, so they do not bother with these trips…(6)

## Discussion

**Figure 1.**
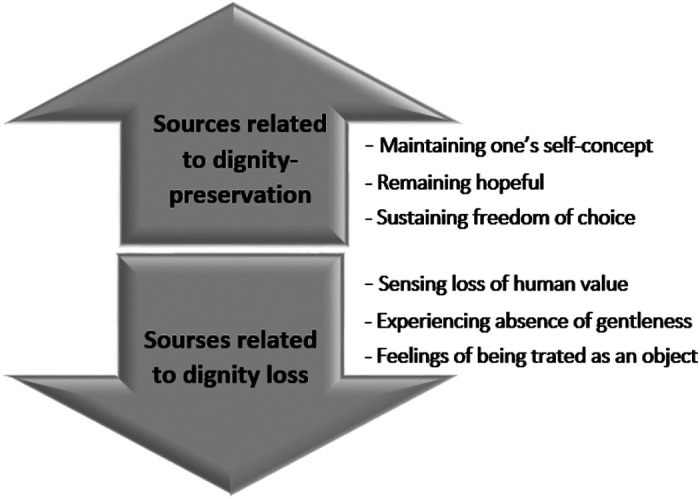
The results divided into categories.

**Figure 2.**
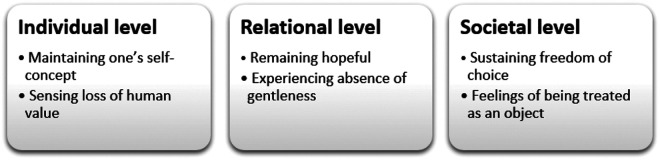
Interpretive understanding of the results on three dignity-related.

Although interrelated, the results presented in Figure 1 are discussed here on three different levels (Figure 2). First, the results highlight sources related to dignity-preservation and dignity loss on the individual level: *maintaining one’s self-concept* and *sensing loss of human value*. Second, the results show that dignity-related experiences of *remaining hopeful* and *experiencing absence of gentleness* are affected by human interactions, and will thus be discussed on the relation level. Finally, we will discuss what we identify as core dignity-related sources on the societal level: *sustaining freedom of choice* and *feelings of being treated as an object.*

On the individual level, being treated with respect and having the opportunity to maintaining one’s self-concept and control in life seemed essential for preserving older women’s dignity experiences. As found in previous studies,^1,27,28^ it was important for the women to feel significant and valuable while living with incurable cancer. Being involved as the person one is and strives to become, is related to dignity.^27^ In work by Benson et al.^28^ ICs highlighted older women’s experiences of dignity as central sources in generating power to help them making their own decisions. Our results support these findings, while adding new nuances; the ICs emphasized the women’s vital need to maintain their individual self in everyday-life situations, whereas common self-care, independently having a shower and preparing meals were examples of coping and finding joy in daily activities. Aiming to continue their lives, treasuring these habits and searching for the bright spots in life, were perceived as vital for upholding their senses of self-worth and personal integrity. Barclay^29^ points out that the patient is maintaining her dignity when she is able to live in accordance with her standards and values. Values and standards can however deteriorate, particularly when vulnerable and living with a life-threatening illness.^29^ These perspectives support the findings of our study, where the ICs reported how the women described unworthy experiences related to getting old and being incurable ill. Becoming weaker both physical and mentally, evoked feelings of meaninglessness and existential loneliness. Despite these degrading experiences, care theorist Katie Eriksson^30^ highlights human equality, describing each human being as *unique* − an entity of body, soul and spirit − even in times of vulnerability. For the women to experience security and meaningfulness, Eriksson^31^ highlights three different living spaces to be optimized: *the physical living space, the psychosocial living space* and *the existential living space.* Eriksson associates the perspective of the concrete place to stay with what she terms the *physical living space*.^31^ The ICs related this to the physical environment, a place where the women could feel safe, valuable and in charge of decisions. In the *psychosocial living space*, all relational interactions between the women and people in their surroundings take place – affecting their everyday experiences. Referring to the *existential living space* Eriksson^31^ describes this as a place where each human being find and nurture the inner thoughts, wishes and hopes that promote a meaningful life. When nearing end-of-life, different circumstances can obstruct these women’s *living spaces*, leading to experiences of dignity loss. It is therefore of vital importance for HCPs to gain knowledge and awareness of these dimensions in order to increase the women’s individual intrapersonal dignity experiences.

On the relational level, dignity-preserving care was experienced when the women felt recognized and confirmed as worthy human beings by the HCPs, being able to take part in conversations and being treated with equality. Previous research reports that the attitude of HCPs and their unwillingness to preserve the patient’s autonomy deprived the women of their standards and values.^29^ These findings also relate to Eriksson’s *Caring culture* ^32^ in which she states that all caring is formed in the relationship between the human being and the caregiver. However, the findings indicate that the attitude of HCPs could negatively affect the women’s experiences of dignity. As observed during participation observations and described by ICs, there were situations where the women felt humiliated due to what they experienced as uncaring behaviours, in particular in situations where the women felt dependent upon HCPs. These findings are similar to the patients’ stories described in Chochinov’s research^33^ where he highlights HCPs’ duty to provide the most comprehensive empathic end-of-life care to relieve suffering and distress. Laursen et al.^34^ support this view and emphasize that patients nearing end-of-life, who experience existential loneliness, have a particular need for HCPs who are dedicated to identifying their patients’ individual needs. They highlight that every human being should be seen and supported in their everyday lives, as this strengthens their senses of dignity.^34^ The findings of our study contribute to this area of research by calling for HCPs to pay attention to the core values of genuine presence and gentleness. Closeness and having a kind and agreeable manner may increase the women’s senses of worthiness, help uphold their experiences of personal integrity and strengthen their *relative dignity* – a modifiable form of dignity described by Eriksson.^24,31^ *Relative dignity* can be preserved when the *suffering human being* receives *caring recognition and confirmation* from others, to use Eriksson’s terms.^31^ Being modifiable, the relative dignity can also be torn down through external humiliation, leading to dignity loss.^31^ Therefore, the caring attitude and behaviour of each HCP is vital to preserve the relative dignity of these women nearing end-of-life.

Importantly, Eriksson^31^ also describes another inalienable form of dignity termed *absolute dignity.* Founded upon this view of human nature, absolute dignity is inherent in all people, granted by virtue of being human. Consequently, recognizing the absolute dignity of each human being is a fundamental view of humanity with implications for caring since this constitutes HCPs core understanding of themselves, the patient, the ethics of caring, and how they themselves affect the relative dignity of their patients. These crucial, relational sources can be seen as vital dimensions and parts of the women’s *psychosocial living space*.^31^ Experiencing the power of relationships happens in this living space, which may confirm the women’s value and relative dignity as well as enhance their senses of control and self-determination.

Finally, on the societal level, the results suggest that the women have a fundamental need to be treated with compassionate understanding in travel situations. In some situations, the women felt regarded as an object, with no significance. According to Fjose et al.,^35^ transportations characterized by discomfort and overfull taxis are exhausting and may even lead to re-admissions. ICs in this present study reported that these old and frail women sometimes considered cancelling treatment appointments at the hospital to avoid dignity-depriving transportation experiences. The ICs description of the older women’s lack of influence on the transportation routines, contrasts the women’s need of sustaining their freedom of choices. With respect to choices, the ICs expressed the importance of giving the women the option to tailor their own end-of-life care, and make decisions concerning the preferable place to stay when nearing end-of-life − even with limited strengths. Autonomy and personal integrity have previously been found to be essential aspects inherent in dignity-preserving care,^36^ and are at stake in situations like these.

The results of this present study also show that the women’s preferences regarding the place to stay when nearing end-of-life were characterized by doubts, insecurities and lack of coherences. The ICs described how the women wanted to stay in familiar surroundings and live their final days in their own, treasured homes. However, when feeling insecure, some women wanted to be admitted to an institution to receive continuity of care and reduce IC burden – in both cases their preferred *physical living space* according to Eriksson.^31^ Importantly, the reciprocal interaction of the individual, relational and societal sources affecting dignity should be recognized. These sources serve to illustrate that dignity can be affected on different levels. In addition, since being interrelated the dignity experience on one level may influence the sense of dignity also on the other levels, or to use Eriksson’s words; *the physical living space, the psychosocial living space* and *the existential living space* are parts of the human living space as a whole.^31^ As reported by ICs and discussed in this study, dignity-preserving care for older, home-dwelling women living with incurable cancer can be promoted when focused attention is given to uphold their living spaces according to their wishes and needs. This knowledge should therefore be emphasized in HCPs’ educational training and in the municipal end-of life care for these patients.

## Methodological considerations

We found in-depth interviews and participant observations to be suitable data-collection tools. The first author’s long practice as a municipal oncology nurse helped her establish a safe and good atmosphere during data collection. Ten ICs declined to participate in the study, and we acknowledge that a greater number of participants would have strengthened the data collection. While collecting data on the ICs’ experiences, reflecting upon our pre-understanding and focusing on collecting detailed descriptions, afforded study transferability.^37^ As part of the methodological approach, we used the framework PAICPAIR part 1^21^ to strengthen study trustworthiness^38^ regarding the credibility and internal validity of the study;^38^ two ICs contributed in all phases of the research process, as members of the steering group and advisory board. Dependability^38^ was sought through a transparent documentation of the research process. A procedure to empower confirmability^38^ was emphasized, in which checking and rechecking all the collected data was performed thoroughly. To strengthen study authenticity,^38^ portraying the IC’s perceptions as formulated by themselves, was highlighted.

## Conclusion and recommendation

According to ICs, older women experience dignity on the individual level when maintaining one’s self-concept and self-worth. The value of remaining hopeful, experiencing joy and being seen as an independent individual, were crucial dignity-preserving sources on the relational level. On the societal level, the ICs highlighted the women’s need for sustaining freedom of choice concerning the place to be when nearing end-of-life.

Sources leading to dignity loss on the individual level were related to the women’s sense of losing their human value. On the relational level, lack of HCP presence and gentleness, led to a sense of humiliation and worthlessness. On the societal level, feelings of being treated as an object were experienced as a dignity-depriving source. The findings strongly suggest a practical value for emphasizing HCPs ethical training and reflections upon dignity-preserving end-of-life care provided by municipal healthcare services. Regardless of the woman’s preferred place to stay when nearing end-of life, the attitude and behaviour of HCPs should consist of gentleness and consideration, as well as compassion and awareness of the women’s condition and subjective needs. Further research should explore dignity and dignity loss as perceived by HCPs responsible for the care and treatment of older women living with incurable cancer at home. Further studies are needed to see how changes in municipal end-of-life care services affect experiences of dignity for this group of patients.
